# A Mathematical Model for the Flow of a Casson Fluid due to Metachronal Beating of Cilia in a Tube

**DOI:** 10.1155/2015/487819

**Published:** 2015-02-19

**Authors:** A. M. Siddiqui, A. A. Farooq, M. A. Rana

**Affiliations:** ^1^Department of Mathematics, Pennsylvania State University, York Campus, York, PA 17403, USA; ^2^Department of Basic Sciences, Riphah International University, Islamabad 44000, Pakistan; ^3^COMSATS Institute of Information Technology, Tobe Camp, Abbottabad 22010, Pakistan

## Abstract

A mathematical model is developed to study the transport mechanism of a Casson fluid flow inspired by the metachronal coordination between the beating cilia in a cylindrical tube. A two-dimensional system of nonlinear equations governing the flow problem is formulated by using axisymmetric cylindrical coordinates and then simplified by employing the long wavelength and low Reynolds number assumptions. Exact solutions are derived for the velocity components, the axial pressure gradient, and the stream function. However, the expressions for the pressure rise and the volume flow rate are evaluated numerically. The features of the flow characteristics such as pumping and trapping are illustrated and discussed with the help of graphs. It is observed that the volume flow rate is influenced significantly by the width of plug flow region *H*_*p*_ as well as the cilia length parameter *ε*. The analysis is also applied and compared with the estimated value of the volume flow rate of epididymal fluid in the ductus efferentes of the human male reproductive tract.

## 1. Introduction

The study of fluid transport due to systems of beating cilia has attracted the attention of many researchers due to its applications in bioengineering and medical sciences. It is generally believed that cilia are responsible for the transport of biological fluids in several physiological processes such as the removal of tracheobronchial mucus in the respiratory track, the transport of ovulatory mucus and ovum in the oviduct of the female reproductive tract system, and the motion of epididymal fluid in the efferent ductus of the human male reproductive tract [1–10]. Failure of the transport functionality of cilia can cause a serious illness of the respiratory system, pathological transport of bacteria, and infertility in human uterus. The mechanism of cilia transport has also been exploited for the design and fabrication of artificial cilia for microfluidic applications [11–13].

Cilia are hair-like appendages extending from the surface of many cells and deform in a wave-like fashion to propel either cell itself or the fluid around it. A ciliated organism carries high densities of cilia arranged in rows along and across the body surface. Cilia beat in a whip-like asymmetric manner consisting of an effective stroke and a recovery stroke. Moreover, when many cilia operate together, hydrodynamic interactions cause them to beat out of phase leading to the formation of metachronal waves and an enhanced fluid flow [1–10]. Like other types of waves, these waves can also be described by the amplitude, wavelength, and the frequency. Ciliated surfaces are known to have different patterns, depending upon whether the metachronal wave travels in the direction of effective stroke, called symplectic metachronism, or in the opposite direction to the effective stroke known as antiplectic metachronism, which is in the opposite direction of fluid motion. Cilia usually operate in an environment of low Reynolds number where the effect of inertia is negligible [1–4].

A survey of the literature shows that Jahn and Bovee [14] studied the hydrodynamics of protozoa which use cilia for locomotion. Later, various scientists have studied this mechanism with considerations of nature of fluids in different flow geometries and now several solutions are available in the literature. The interested reader can see the literature regarding the cilia-induced flows in [1–16]. However, the available information indicates that only a little work has been done to study the role of metachronal wave beating due to active dynamic of cilia on the propulsion of biological fluids through tubules. The motivation of this study comes through a desire to understand the transport of spermatic fluid through the ductus efferentes of the male reproductive tract caused by cilia motion. In 1972, Lardner and Shack [1] developed a model for the flow of a Newtonian viscous fluid due to ciliary activity in the ductus efferentes of the male reproductive tube. They used an envelope over the oscillating cilia to model the metachronal wave. Later on, this approach is used to study the flows of non-Newtonian power law fluids in a ciliated channel with the consideration of different geometries [4–8]. In this study, we have extended the work of Lardner and Shack for a non-Newtonian Casson fluid in an axially symmetric tube for more realistic results. It is now well known that most of the physiological fluids behave like non-Newtonian fluids. In the recent years, several models of non-Newtonian fluids have been proposed by various scientists to investigate the flow behavior in certain physiological systems of living bodies. This is due to their different rheological characteristics. Among these models Casson model is a non-Newtonian fluid model with yield stress and has been widely used for modeling certain biological fluids [17–21]. The cilia transport of Casson fluid in a uniform tube has not been attempted so far. Keeping this fact in mind, we are interested to investigate the flow of Casson fluid in a cylindrical tube due to metachronal wave movement of cilia. The governing equations and the relevant boundary conditions are formulated in an axisymmetric cylindrical coordinate system. The equations are then simplified by using long wavelength approximation in an environment of low Reynolds number. The relationship between pressure rise and the volume flow rate is obtained explicitly. The pumping characteristics and trapping phenomena of ciliary activity are also discussed in detail. A comparison of the results for the volume flow rate of the proposed model to the estimated values of the flow of seminal fluid in the ductus efferentes of the human male reproductive tract system is also given. The graphical behavior of different flow quantities of the mechanism has also been examined for various parameters of interest.

The basic motivation of this study is the hope that such a problem will be helpful in many biomedical as well as industrial applications especially in the study of infertility problems in humans and in the manufacturing of micropumps for drug-delivery systems. Ciliary pumping mechanism may be utilized in the manufacturing of swimming microrobots for biomedical applications [13]. This analysis also offers very interesting applications for the flow control in lab-on-a-chip devices and in tiny biosensors.

## 2. Mathematical Formulation of the Problem

**Figure 1:**
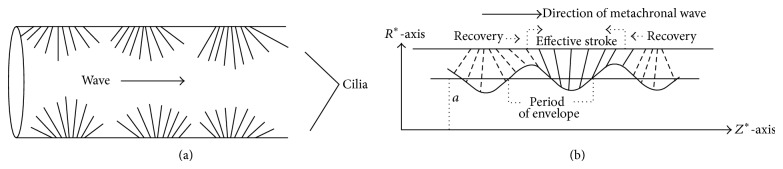
Wave motion due to cilia: (a) ciliated tube and (b) metachronal wave pattern.

Consider the axially symmetric flow of an incompressible Casson fluid in a uniform cylindrical tube whose inner surface is ciliated (see Figure 1). When cilia at the inner surface of the tube operate together, hydrodynamic interactions cause them to beat out of phase leading to the formation of metachronal waves and an enhanced fluid flow. We want to study the fluid transport characteristics of the Casson fluid in the tube as a function of cilia and the metachronal wave velocity. Let us choose a cylindrical coordinate system, (*R* ^*^, *Z* ^*^), where *Z* ^*^-axis lies along the centerline of the tube and *R* ^*^-axis is normal to it.

The constitutive equation (relationship between the shear stress and strain rate) of a Casson fluid model may be defined in a simplified form as [17–21]

(1) $$\begin{array}{c} \sqrt{\tau^{*}}=\sqrt{\mu {\dot{\gamma }}^{*}}+\sqrt{\tau_{0}^{*}},\;\text{for}\;\;\tau^{*}\geq \tau_{0}^{*},\\ {\dot{\gamma }}^{*}=0,\;\text{for}\;\;\tau^{*}\leq \tau_{0}^{*}, \end{array}$$

where *τ* ^*^ is the shear stress, *μ* is the viscosity coefficient of Casson fluid, ${\dot{\gamma }}^{*}$ is the rate of shear strain, and *τ*_0_^*^ is the yield stress.

The fundamental equations governing the axially symmetric flow of an incompressible fluid are given by

(2) $$\begin{array}{c} \frac{\partial U^{*}}{\partial R^{*}}+\frac{U^{*}}{R^{*}}+\frac{\partial W^{*}}{\partial Z^{*}}=0,\\ \rho \left[\frac{\partial U^{*}}{\partial t^{*}}+U^{*}\frac{\partial U^{*}}{\partial R^{*}}+W^{*}\frac{\partial U^{*}}{\partial Z^{*}}\right]=-\frac{\partial P^{*}}{\partial R^{*}}+\nabla^{*}\cdot \tau^{*},\\ \rho \left[\frac{\partial W^{*}}{\partial t^{*}}+U^{*}\frac{\partial W^{*}}{\partial R^{*}}+W^{*}\frac{\partial W^{*}}{\partial Z^{*}}\right]=-\frac{\partial P^{*}}{\partial Z^{*}}+\nabla^{*}\cdot \tau^{*}, \end{array}$$

where *U* ^*^ and *W* ^*^ are the velocity components in radial and axial directions, respectively, *P* ^*^ is the pressure, *ρ* is the fluid density, and *τ* ^*^ is the shear stress. Keeping the view on the geometry of the metachronal wave pattern, we assume that the envelope of cilia tips can be expressed mathematically in the following form [1–3]:

(3) $$\begin{array}{c} R^{*}=H^{*}=f^{*}\left(Z^{*},t^{*}\right)=a+a\varepsilon \cos\left(\frac{2\pi }{\lambda }\left(Z^{*}-ct^{*}\right)\right), \end{array}$$

which also describes the equation of an extensible boundary for the flow in the tube. Here *a* is the mean radius of the ciliated tube, *ε* is a nondimensional measure with respect to *a*, the cilia length, and *λ* and *c* denote the wave length and wave speed of the metachronal wave. Based upon different patterns of cilia beating observed by Sleigh [2, 3], the cilia tips can be considered to move in elliptical paths such that the horizontal positions of the cilia tips can be given by

(4) $$\begin{array}{c} Z^{*}=g^{*}\left(Z^{*},Z_{0}^{*},t^{*}\right)=Z_{0}^{*}+a\varepsilon \alpha \sin\left(\frac{2\pi }{\lambda }\left(Z^{*}-ct^{*}\right)\right), \end{array}$$

where *Z*_0_^*^ is some reference position of the particle and *α* is a measure of the eccentricity of the elliptical motion of the cilia tips. If no slip condition applies, the velocities imparted to the fluid particles are just those of the cilia tips and hence the axial and radial velocities of the cilia can be formulated as

(5) $$\begin{array}{c} W^{*}={\left.\frac{\partial Z^{*}}{\partial t^{*}}\right|}_{Z_{0}^{*}}=\frac{\partial g^{*}}{\partial t^{*}}+\frac{\partial g^{*}}{\partial Z^{*}}\frac{\partial Z^{*}}{\partial t^{*}}=\frac{\partial g^{*}}{\partial t^{*}}+\frac{\partial g^{*}}{\partial Z^{*}}W^{*},\\ U^{*}={\left.\frac{\partial R^{*}}{\partial t^{*}}\right|}_{Z_{0}^{*}}=\frac{\partial f^{*}}{\partial t^{*}}+\frac{\partial f^{*}}{\partial Z^{*}}\frac{\partial Z^{*}}{\partial t^{*}}=\frac{\partial f^{*}}{\partial t^{*}}+\frac{\partial f^{*}}{\partial Z^{*}}W^{*}. \end{array}$$

Using (3) and (4) into (5) and solving them, one can easily obtain

(6) $$\begin{array}{c} W^{*}=\frac{-\left(2\pi /\lambda \right)\left(\varepsilon \alpha ac\cos\left(2\pi /\lambda \right)\left(Z^{*}-ct^{*}\right)\right)}{1-\left(2\pi /\lambda \right)\left(\varepsilon \alpha a\cos\left(2\pi /\lambda \right)\left(Z^{*}-ct^{*}\right)\right)},\\ U^{*}=\frac{\left(2\pi /\lambda \right)\left(\varepsilon ac\sin\left(2\pi /\lambda \right)\left(Z^{*}-ct^{*}\right)\right)}{1-\left(2\pi /\lambda \right)\left(\varepsilon \alpha a\cos\left(2\pi /\lambda \right)\left(Z^{*}-ct^{*}\right)\right)}. \end{array}$$

We require *W* ^*^ and *U* ^*^ to be the axial and radial velocities on the boundary of the flow domain given in (3).

The present investigation will be carried out in the coordinate system (*r* ^*^, *z* ^*^) moving with respect to the fixed coordinate system (*R* ^*^, *Z* ^*^), in which the boundary shape (3) is stationary. These two coordinate systems are related by the expressions

(7) z∗=Z∗−ct∗,  r∗=R∗,  w∗=W∗−c,u∗=U∗,  p∗z∗,r∗=P∗Z∗,R∗,t∗,

in which (*w* ^*^, *u* ^*^, *p* ^*^) and (*W* ^*^, *U* ^*^, *P* ^*^) are the velocity components and the pressure in the moving and the fixed coordinates, respectively.

We introduce the following nondimensional quantities:

(8) r=r∗a,  z=z∗λ,  u=u∗βc,  w=w∗c,β=aλ,  p=a2p∗cμλ,  h=H∗a,  Re=ρcaμβ,τ=aτ∗μc,  τ0=aτ0∗μc,  γ˙=aγ˙∗c,q=q∗2πa2c,  Q=Q∗2πa2c,

where *Re* is the modified Reynolds number, *α* is the measure of eccentricity of the elliptical motion, *β* is the wave number, *ε* is a dimensionless parameter representing the cilia length, and *q* and *Q* both stand for the dimensionless volume flow rates.

After using the above nondimensional parameters and then employing the assumptions of long wavelength and low Reynolds number, the equations governing the flow of Casson fluid can be reduced to the following forms:

(9) $$\begin{array}{c} \sqrt{\tau }=\sqrt{\tau_{0}}+\sqrt{\frac{\partial w}{\partial r}},\;\text{for}\;\;\tau \geq \tau_{0}, \end{array}$$

(10) $$\begin{array}{c} \frac{\partial w}{\partial r}=0,\;\text{for}\;\;\tau \leq \tau_{0}, \end{array}$$

(11) $$\begin{array}{c} \frac{1}{r}\frac{\partial \left(ru\right)}{\partial r}+\frac{\partial w}{\partial z}=0, \end{array}$$

(12) $$\begin{array}{c} \frac{\partial p}{\partial z}=\frac{1}{r}\frac{\partial \left(r\tau \right)}{\partial r}, \end{array}$$

(13) $$\begin{array}{c} \frac{\partial p}{\partial r}=0, \end{array}$$

(14) $$\begin{array}{c} r=h=1+\varepsilon \cos\left(2\pi z\right). \end{array}$$

The following dimensionless boundary conditions are imposed on the governing equations as follows:

 
*no slip condition on the inner surface of the tube, that is,*
  (15) $$\begin{array}{c} w\left(z,h\right)=w\left(h\right)=-1-2\pi \varepsilon \alpha \beta \cos\left(2\pi z\right), \end{array}$$
 
*radial velocity at the wall of the tube, that is,*
  (16) uz,h=u(h)=2πεsin(2πz)+αβ2πε2sin2πzcos⁡2πz,
 
*absence of any radial velocity in the plug flow region, that is,*
  (17) $$\begin{array}{c} u\left(z,H_{p}\right)=0, \end{array}$$
 
*regularity condition, that is,*
  (18) $$\begin{array}{c} \frac{\partial w}{\partial r}\left(z,H_{p}\right)=0, \end{array}$$

where *H*_*p*_ is the radius of the plug flow region and it is defined by

(19) $$\begin{array}{c} H_{p}=\frac{2\tau_{0}}{\partial p/\partial z}. \end{array}$$

It is interesting to note that when *τ*_0_ = 0  (*H*_*p*_ = 0), (9)–(19) reduce to those for simple Newtonian fluid [1] and when *τ*_0_ = 0 and *α* = 0, the results of Shapiro et al. [22] can be achieved.

## 3. Solution and the Flow Analysis

Equation (13) indicates that the pressure *p* is not a function of *r*. This enables us to use the total derivative *dp*/*dz* in (12). Now integrating (12) once with respect to *r*, we obtain

(20) $$\begin{array}{c} \tau =\frac{C}{r}+\frac{r}{2}\frac{dp}{dz}, \end{array}$$

where *C* is a constant of integration.

Making use of (20) into (9) along with the boundary condition (18), we get

(21) $$\begin{array}{c} \frac{\partial w}{\partial r}=\frac{1}{2}\frac{dp}{dz}\left\{r+H_{p}-2\sqrt{rH_{p}}\right\}, \end{array}$$

whose solution satisfying the boundary condition (15) can be written as

(22) w=w(h)+14dpdz(r−h)(r+h+2Hp)−83Hpr3/2−h3/2.

Substituting *r* = *H*_*p*_ in (22), we get the velocity in the plug flow region as

(23) wp=w(h)+14dpdz·Hp−hh+3Hp−83HpHp3/2−h3/2.

It is noted that (22) gives the expression for axial velocity of the fluid in a moving coordinate system in terms of the pressure gradient. As in the theory of lubrication this pressure gradient has to be derived from an expression for the volume flow rate which is constant in the moving coordinate system. So

(24) $$\begin{array}{c} q=2\overset{h}{\underset{0}{\int }}rwdr=2\overset{H_{p}}{\underset{0}{\int }}rw_{p}dr+2\overset{h}{\underset{H_{p}}{\int }}rwdr. \end{array}$$

Substituting the expressions for *w*_*p*_ and *w* in the above equation and after simplification, we get *dp*/*dz* in terms of constant volume flow rate *q*, as

(25) $$\begin{array}{c} \frac{dp}{dz}=\frac{h^{2}w\left(h\right)-q}{h^{3}\left[\left(1/24\right)\left(3h+4H_{p}\right)-\left(2\sqrt{hH_{p}}/7\right)\right]-\left(H_{p}^{4}/168\right)}. \end{array}$$

For one wavelength of the metachronal wave, the integration of (25) yields the expression of pressure rise Δ*p* in the following form:

(26) $$\begin{array}{c} \Delta p=\overset{1}{\underset{0}{\int }}\frac{dp}{dz}dz=I_{1}-qI_{2}, \end{array}$$

where

(27) $$\begin{array}{c} I_{1}=\overset{1}{\underset{0}{\int }}\frac{h^{2}w\left(h\right)dz}{h^{3}\left[\left(1/24\right)\left(3h+4H_{p}\right)-\left(2\sqrt{hH_{p}}/7\right)\right]-\left(H_{p}^{4}/168\right)},\\ I_{2}=\overset{1}{\underset{0}{\int }}\frac{dz}{h^{3}\left[\left(1/24\right)\left(3h+4H_{p}\right)-\left(2\sqrt{hH_{p}}/7\right)\right]-\left(H_{p}^{4}/168\right)}. \end{array}$$

It is noticed that the integrals *I*_1_ and *I*_2_ cannot be integrated in closed form and therefore are evaluated numerically by using a Mathematics Software Maple.

The constant flux *q* is related to the dimensionless volume flow rate *Q* through the following relation:

(28) $$\begin{array}{c} Q=2\overset{h}{\underset{0}{\int }}r\left(w+1\right)dr=q+h^{2}. \end{array}$$

The dimensionless time-mean volume flow rate $\overset{-}{Q}$ is obtained by using (28) as

(29) $$\begin{array}{c} \overset{-}{Q}=\frac{1}{T}\overset{T}{\underset{0}{\int }}\overset{h}{\underset{0}{\int }}2r\left(w+1\right)drdt=q+1+0.5\varepsilon^{2}, \end{array}$$

where *q* is given by (25). With the help of (26) and (29), the expression for Δ*p* turned out to be

(30) $$\begin{array}{c} \Delta p=I_{1}-\left(\overset{-}{Q}-1-0.5\varepsilon^{2}\right)I_{2}. \end{array}$$

Equation (30) is rewritten in the form

(31) $$\begin{array}{c} \overset{-}{Q}=1+0.5\varepsilon^{2}+\frac{I_{1}}{I_{2}}-\frac{\Delta p}{I_{2}}. \end{array}$$

The corresponding stream function (*w* = (1/*r*)(∂*ψ*/∂*r*), *u* = −(1/*r*)(∂*ψ*/∂*z*)) for the flow under consideration can be written as

(32) ψ=14h2w(h)−Q−−1−(1/2)ε2(r4/4)−(r2h2/2)+2Hpr3/3−r2h2/2−(16/21)Hpr7/2−(7/4)r2h3/2h3(1/24)3h+4Hp−(2hHp/7)−(Hp4/168)   +2r2w(h)h2w(h)−Q−−1−(1/2)ε2(r4/4)−(r2h2/2)+2Hpr3/3−r2h2/2−(16/21)Hpr7/2−(7/4)r2h3/2h3(1/24)3h+4Hp−(2hHp/7)−(Hp4/168).

## 4. Discussion of the Results

**Figure 2:**
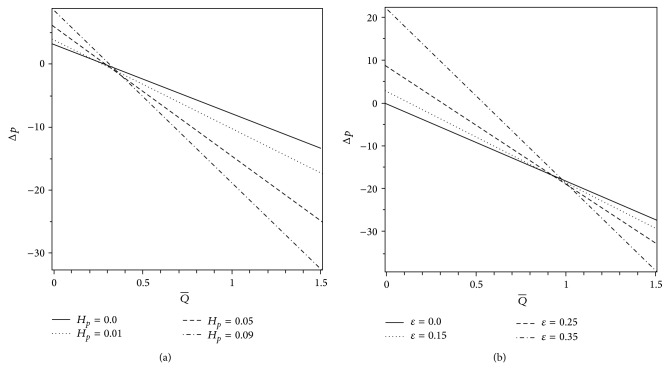
Variations of Δ*p* with $\overset{-}{Q}$ for different values of (a) *H*_*p*_ when *ε* = 0.25 and (b) *ε* when *H*_*p*_ = 0.09. The other parameters are *α* = 0.4 and *β* = 0.4.

**Figure 3:**
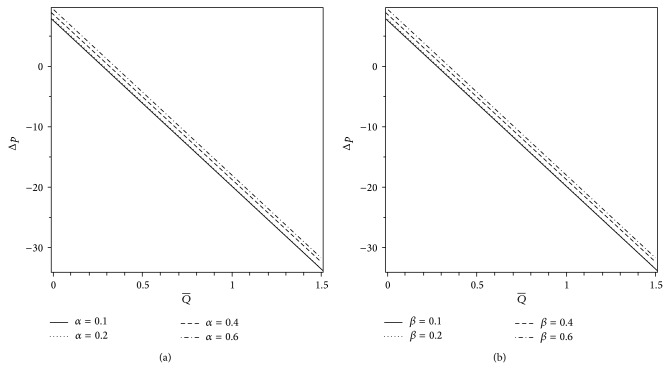
Variations of Δ*p* with $\overset{-}{Q}$ for different values of (a) *α* when *β* = 0.4 and (b) *β* when *α* = 0.4. The other parameters are *H*_*p*_ = 0.09 and *ε* = 0.25.

**Figure 4:**
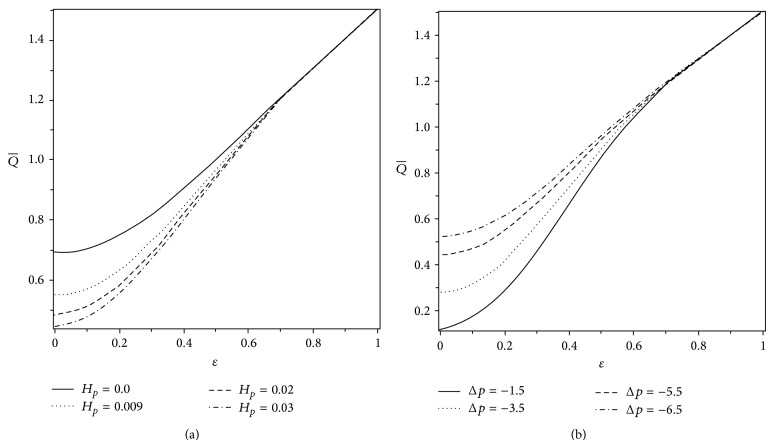
Variations of $\overset{-}{Q}$ with *ε* for different values of (a) *H*_*p*_ when Δ*p* = −5.5 and (b) Δ*p* when *H*_*p*_ = 0.03. The other parameters are *α* = 0.4 and *β* = 0.4.

**Figure 5:**
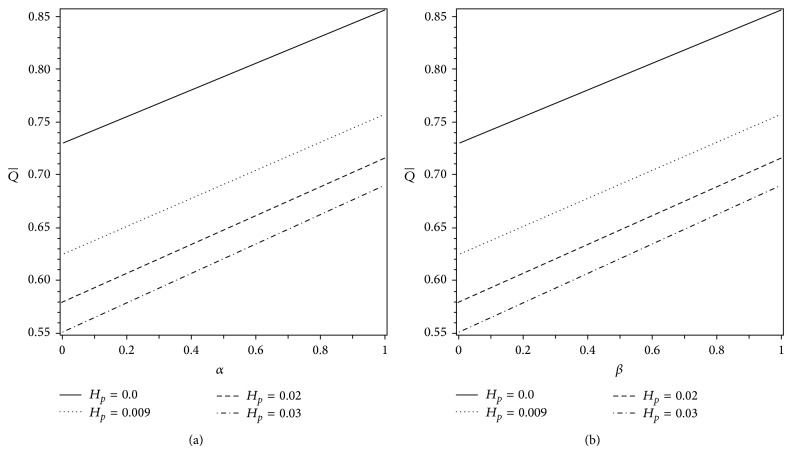
Variations of $\overset{-}{Q}$ for different values of *H*_*p*_ with (a) *α* when *β* = 0.4 and (b) *β* when *α* = 0.4. The other parameters are *ε* = 0.25 and Δ*p* = −5.5.

In this section, we provide a careful analysis of the pressure rise per wavelength Δ*p*, the volume flow rate $\overset{-}{Q}$, the axial pressure gradient *dp*/*dz*, the axial velocity profile *w*, and the streamlines with the help of graphs. Pressure rise per wavelength is an important physical measure in ciliary pumping mechanism. Therefore, the variation of Δ*p* versus the volume flow rate $\overset{-}{Q}$ is shown in Figures 2 and 3 for different values of the plug flow width *H*_*p*_, the cilia length parameter *ε*, the eccentricity parameter of the elliptical motion *α*, and the wave number *β*. It is evident from these graphs that there is an inversely linear relation between Δ*p* and $\overset{-}{Q}$; that is, an increase in the flow rate reduces the pressure rise and vice versa. From Figure 2(a), we can easily observe that there is a critical value of the volume flow rate, approximately $\overset{-}{Q}=0.3$, below which the pressure rise is positive and above which the pressure rise is negative. This value of the volume flow rate is known as free pumping flux. Furthermore, we observe that an increase in the plug flow width *H*_*p*_ causes an increase in the magnitude of the pressure rise Δ*p*. This is a revelation that the pumping machinery has to function more efficiently to push ahead a Casson fluid (*H*_*p*_ ≠ 0) in comparison to a Newtonian fluid (*H*_*p*_ = 0).  Figure 2(b) shows that the pressure rise Δ*p* increases with an increase in the cilia length parameter *ε* until a critical value of the volume flow rate is achieved, approximately $\overset{-}{Q}=0.98$, and thereafter an opposite behavior of Δ*p* is observed. Therefore, by choosing suitable values of $\overset{-}{Q}$ and *ε*, someone may enhance the pumping rate (Δ*p* versus $\overset{-}{Q}$). The effects of *α* and *β* on Δ*p* versus the volume flow rate $\overset{-}{Q}$ (i.e., the pumping rate) are shown in Figures 3(a) and 3(b). It is noticed that the pumping rate increases with increasing *α* and *β* in the pumping (Δ*p* > 0) as well as copumping (Δ*p* < 0) regions. Figures 4(a) and 4(b) exhibit the effects of *H*_*p*_ and Δ*p* on the volume flow rate $\overset{-}{Q}$ versus the cilia length parameter *ε*. It is observed that $\overset{-}{Q}$ decreases with an increase in *H*_*p*_ while it increases when Δ*p* decreases. However, the variations of $\overset{-}{Q}$ with *H*_*p*_ and Δ*p* are found to be insignificant for large values of *ε*. Figures 5(a) and 5(b) display the graphs of volume flow rate $\overset{-}{Q}$ versus *α* and *β* for different values of *H*_*p*_. As expected, the volume flow rate increases linearly with *α* and *β* while it decreases when we increase the width of the plug flow region.

**Figure 6:**
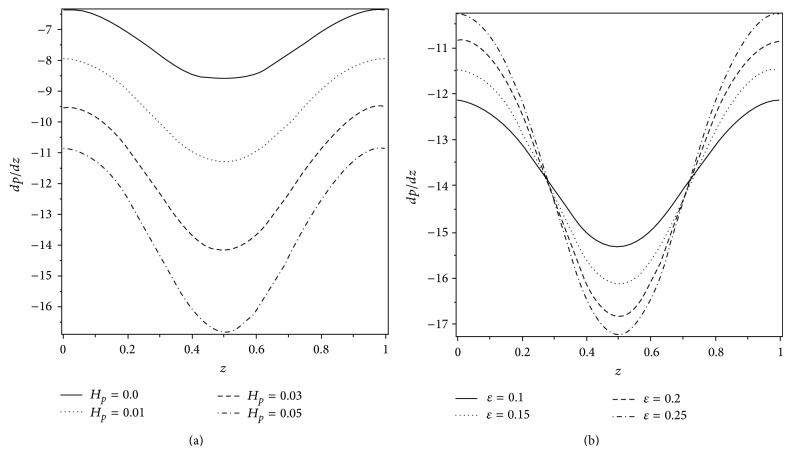
Variation of axial pressure gradient *dp*/*dz* for different values of (a) *H*_*p*_ when *ε* = 0.2 and (b) *ε* when *H*_*p*_ = 0.05. The other parameters are *α* = 0.4, *β* = 0.4, and $\overset{-}{Q}=0.95$.

**Figure 7:**
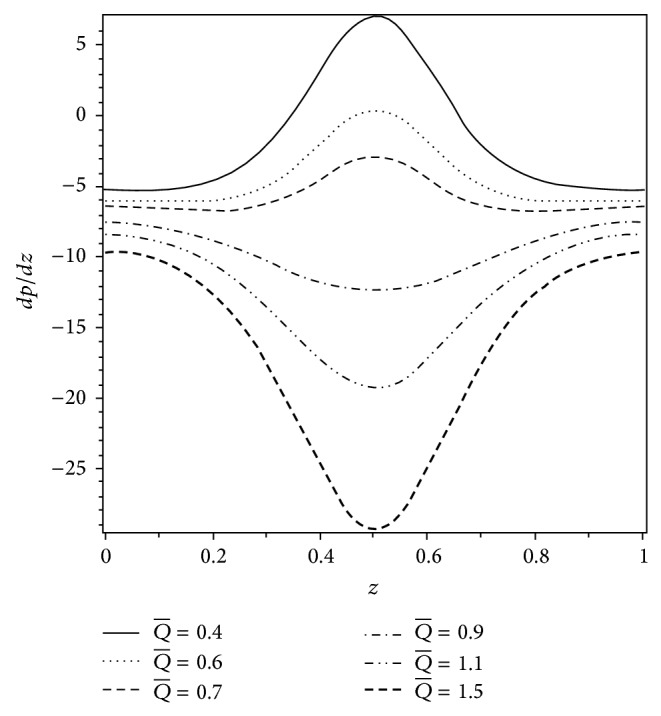
Variation of axial pressure gradient *dp*/*dz* for different values of $\overset{-}{Q}$ when *H*_*p*_ = 0.05, *ε* = 0.25, *α* = 0.4, and *β* = 0.4.

Figures 6(a) and 6(b) are prepared to see the effects of *H*_*p*_ and *ε* on the axial pressure gradient *dp*/*dz*. We observe that the amplitude of the pressure gradient increases with the increasing values of *H*_*p*_ and *ε* and *dp*/*dz* is maximum at *z* = 0.5. It is also seen that, in the narrow part of the *z*-region, the pressure gradient is relatively large; that is, the fluid requires a large amount of pressure gradient to pass through the region. Furthermore, the magnitude of the pressure gradient is smaller for the Newtonian fluid as compared to the Casson fluid. This supports the inference that a large magnitude of *dp*/*dz* is required to maintain the same flux of Casson fluid in comparison to the Newtonian fluid. The effect of volume flow rate $\overset{-}{Q}$ on the axial pressure gradient is shown in Figure 7. It is observed that *dp*/*dz* is positive when $\overset{-}{Q}<0.6$. In this situation, the pressure gradient is not favorable for the flow and is known as adverse pressure gradient. But when $\overset{-}{Q}>0.6$, pressure gradient is negative and is called favorable pressure gradient. In this case, *dp*/*dz* assists the flow in the channel.

**Figure 8:**
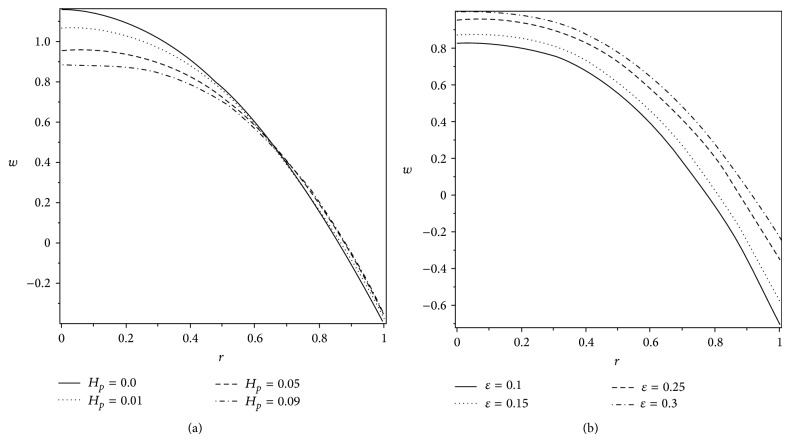
Variation of axial velocity *w* at *z* = 1 for different values of (a) *H*_*p*_ when *ε* = 0.25 and (b) *ε* when *H*_*p*_ = 0.05. The other parameters are *α* = 0.4, *β* = 0.4, and $\overset{-}{Q}=0.95$.

**Figure 9:**
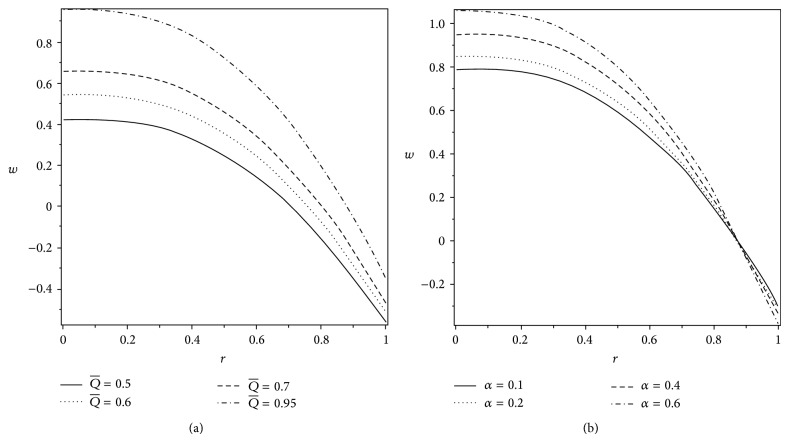
Variation of axial velocity *w* at *z* = 1 for different values of (a) *α* when $\overset{-}{Q}=0.95$ and (b) $\overset{-}{Q}$ when *α* = 0.4. The other parameters are *H*_*p*_ = 0.05, *β* = 0.4, and *ε* = 0.25.

The axial velocity profile *w* is plotted in Figures 8 and 9 for various values of *H*_*p*_, *ε*, $\overset{-}{Q}$, and *α*. It is noted from Figure 8 that *w* gets lessened with the increase in the plug flow region *H*_*p*_ in the part 0 ≤ *r* ≤ 0.58, but in the remaining part, the velocity rises with *H*_*p*_. However, the velocity increases in the whole region with an increase in the cilia length parameter *ε*.  Figure 9(a) indicates that, with the increase in the volume flow rate, the velocity distribution increases in the whole region. But when we look at Figure 9(b), we can describe that the velocity increases with *α* in the part 0 ≤ *r* ≤ 0.83 and decreases in the remaining part with an increase in *α*.

**Figure 10:**
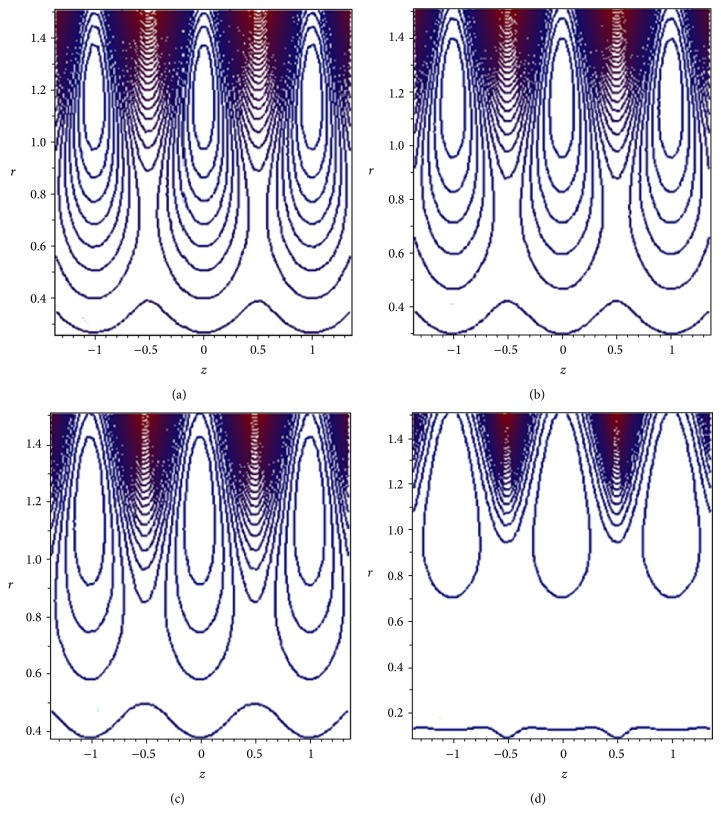
Streamlines for (a) *H*_*p*_ = 0.0, (b) *H*_*p*_ = 0.01, (c) *H*_*p*_ = 0.05, and (d) *H*_*p*_ = 0.09. The other parameters are *ε* = 0.25, *α* = 0.4, *β* = 0.4, and $\overset{-}{Q}=0.95$.

**Figure 11:**
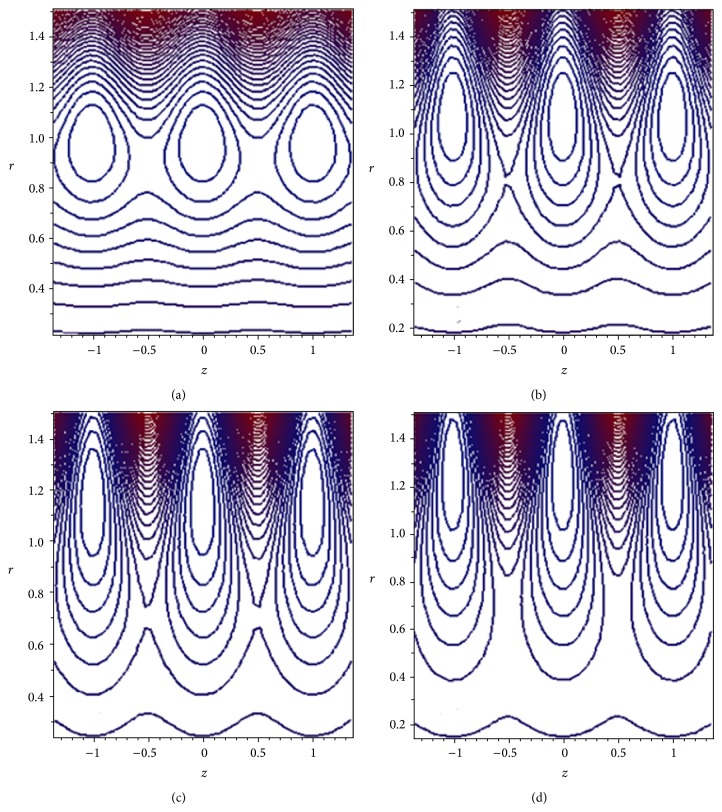
Streamlines for (a) *ε* = 0.05, (b) *ε* = 0.15, (c) *ε* = 0.25, and (d) *ε* = 0.35. The other parameters are *H*_*p*_ = 0.05, *α* = 0.4, *β* = 0.4, and $\overset{-}{Q}=0.95$.

Another interesting phenomenon in the cilia transport is trapping. In the wave frame, streamlines under certain conditions split to trap a bolus of fluid which moves as a whole with the speed of metachronal wave. The effect of the plug flow width *H*_*p*_ on the streamlines pattern is demonstrated through Figure 10. It is observed that the size and the number of closed streamlines trapping boluses reduce as we increase the width of the plug flow region. The influence of the cilia length parameter *ε* is illustrated in Figure 11. It is found that as *ε* increases, the size and the number of closed streamlines trapping boluses increase. Thus, the trapping is opposed by the presence of yield stress and is favored by the cilia length parameter.

## 5. Application: Fluid Transport in the Ductus Efferentes

We have formulated a mathematical model to study the fluid transport characteristics in an axisymmetric tube under the action of ciliary beat that generates a metachronal wave. This type of fluid transport is observed in the ductus efferentes of the human male reproductive tract. The ductuli efferentes in human body are usually 10–15 tubules connecting the rete testis to the epididymis. The cells lining these tubules are ciliated and are responsible for the transport of fluids. As pointed out by Lardner and Shack [1], the approximate value of the flow rate in human rete testis per ductus efferentes can be estimated as 6 × 10^−3^ mL h^−1^ with approximate dimensions of *a* = 50 *μ*m, with frequency of beat of the cilia being 20 sec^−1^ and *c* = (20 beats sec^−1^) × 10 *μ* = 200 *μ*sec^−1^. These values justify the use of long wavelength and low Reynolds number approximations in this analysis. Lardner and Shack [1] calculated the approximate values of nondimensional flow rate $\overset{-}{Q}$ and the dimensional flow rate *Q* ^*^($=\pi a^{2}c\overset{-}{Q}$) as 2.2 × 10^−2^ and 0.12 × 10^−3^ mL h^−1^, respectively, by using *ε* = 0.1, *β* = 0.1, *α* = 1, and Δ*p* = 0 in their model. Unfortunately, the calculated value of *Q* ^*^ is not in good agreement with the experimentally estimated value. However, if we choose *ε* = 0.3, *β* = 0.1, and Δ*p* = −5.5 in our model, we obtain, for a Newtonian fluid (*H*_*p*_ = 0),

(33) $$\begin{array}{c} \overset{-}{Q}=1.110356,\;\;Q^{*}=0.006278. \end{array}$$

For non-Newtonian fluid (*H*_*p*_ = 0.01),

(34) $$\begin{array}{c} \overset{-}{Q}=1.07273,\;\;Q^{*}=0.00606, \end{array}$$

which appears to be the most favorable result. In the end, we hope that the present analysis is useful from biomedical point of view as not much information on this topic is currently available. We also believe that considerably more theoretical and experimental investigations are necessary to understand adequately the mechanism involved in the transport of semen in the ductus efferentes.

## 6. Concluding Remarks

In the present analysis, we have examined the role of cilia motion in terms of metachronal waves in the transport of a Casson fluid through an axially symmetric tube. The implication of long wavelength and low Reynolds number allows us to obtain the flow exactly. The main findings of the above analysis may be summarized as follows.

- It is noted that the relation between Δ*p* and $\overset{-}{Q}$ for the Casson fluid is linear (as for Newtonian fluid). Also, the pressure difference required to refrain the flow completely is positive.
- The magnitude of the pressure rise increases with an increase in the plug flow width which shows that the pumping rate decreases for the Casson fluid in comparison to the Newtonian fluid.
- Δ*p* and $\overset{-}{Q}$ are showing opposite behaviors for all values of other parameters.
- The pressure gradient required to pass the same amount of a Casson fluid is comparatively larger than that of a Newtonian fluid under the same set of conditions.
- It is observed that, with an increase in *H*_*p*_, the velocity distribution decreases with an increase in the plug flow width in the central part of tube but increases near the boundary. However, the effect of *H*_*p*_ on the velocity distribution near the boundary is not much significant.
- The size and the number of circulations of the closed streamlines reduce as we increase the width of plug flow region.
- It is found that the calculated value of the volume flow rate by using our model is 0.00606 mL h^−1^ and is in excellent agreement with the estimated value as pointed out in [1].
- The corresponding results for a Newtonian fluid can be recovered as a special case from our results by taking *H*_*p*_ = 0.
